# Supplementary material for: HIVprotI: an integrated web based platform for prediction and design of HIV proteins inhibitors
Source: J Cheminform. 2018 Mar 9;10:12. doi: 10.1186/s13321-018-0266-y (PMC5845081; doi:10.1186/s13321-018-0266-y)
Supplement: Supplementary file 2 — Additional file 2. Source code of HIVProtI web server. [file 13321_2018_266_MOESM2_ESM.zip › HIVprotI_Source-code/hivprotI/weka-3-6-6/doc/weka/gui/treevisualizer/package-summary.html]

weka.gui.treevisualizer


---


|  |  |  |
| --- | --- | --- |
| **Overview** \| **Package** \| Class \| **Tree** \| **Deprecated** \| **Index** \| **Help** \| **Weka's home** | |  |
| **PREV PACKAGE**   **NEXT PACKAGE** | **FRAMES**    **NO FRAMES**     **All Classes** |  |


---

## Package weka.gui.treevisualizer

| **Interface Summary** | |
| --- | --- |
| **NodePlace** | This is an interface for classes that wish to take a node structure and arrange them |
| **TreeDisplayListener** | Interface implemented by classes that wish to recieve user selection events from a tree displayer. |

| **Class Summary** | |
| --- | --- |
| **Colors** | This class maintains a list that contains all the colornames from the dotty standard and what color (in RGB) they represent |
| **Edge** | This class is used in conjunction with the Node class to form a tree structure. |
| **Messages** | Messages. |
| **NamedColor** | This class contains a color name and the rgb values of that color |
| **Node** | This class records all the data about a particular node for displaying. |
| **PlaceNode1** | This class will place the Nodes of a tree. |
| **PlaceNode2** | This class will place the Nodes of a tree. |
| **TreeBuild** | This class will parse a dotty file and construct a tree structure from it with Edge's and Node's |
| **TreeDisplayEvent** | An event containing the user selection from the tree display |
| **TreeVisualizer** | Class for displaying a Node structure in Swing. |

---


|  |  |  |
| --- | --- | --- |
| **Overview** \| **Package** \| Class \| **Tree** \| **Deprecated** \| **Index** \| **Help** \| **Weka's home** | |  |
| **PREV PACKAGE**   **NEXT PACKAGE** | **FRAMES**    **NO FRAMES**     **All Classes** |  |


---
